# Kinematic Constrained RRT Algorithm with Post Waypoint Shift for the Shortest Path Planning of Wheeled Mobile Robots

**DOI:** 10.3390/s24216948

**Published:** 2024-10-29

**Authors:** Sisi Liu, Zhan Zhao, Jun Wei, Qianqian Zhou

**Affiliations:** School of Agricultural Engineering, Jiangsu University, Zhenjiang 212013, China; 2212116003@stmail.ujs.edu.cn (S.L.); 2112116018@stmail.ujs.edu.cn (J.W.); 2112216013@stmail.ujs.edu.cn (Q.Z.)

**Keywords:** rapidly exploring random tree, kinematic constraints, wheeled mobile robots, post waypoint shift, shortest path planning

## Abstract

This paper presents a rapidly exploring random tree (RRT) algorithm with an effective post waypoint shift, which is suitable for the path planning of a wheeled mobile robot under kinematic constraints. In the growth of the exploring tree, the nearest node that satisfies the kinematic constraints is selected as the parent node. Once the distance between the new node and the target is within a certain threshold, the tree growth stops and a target connection based on minimum turning radius arc is proposed to generate an initial complete random path. The most significant difference from traditional RRT-based methods is that the proposed method optimizes the path based on Dubins curves through a post waypoint shift after a random path is generated, rather than through parent node selection and rewiring during the exploring tree growth. Then, it is proved that the method can obtain an optimal path in terms of the shortest length. The optimized path has good convergence and almost does not depend on the state of the initial random path. The comparative test results show that the proposed method has significant advantages over traditional RRT-based methods in terms of the sampling point number, the tree node number, and the path node number. Subsequently, an efficient method is further proposed to avoid unknown obstacles, which utilizes the original path information and thus effectively improves the new path planning efficiency. Simulations and real-world tests are carried out to demonstrate the effectiveness of this method.

## 1. Introduction

Planning a collision-free path for specific tasks in an obstacle-prone environment is one of the fundamental task in robotics. For decades, various planning algorithms have been proposed and applied successfully in different applications [1]. Due to the requirements of robots in improving working performance, path optimization has always been an important issue [2]. To achieve different objectives, many optimization criteria have been proposed, such as shortest length [3], path smoothness [4], and least energy consumption [5]. In addition, a feasible path needs to satisfy the robot’s kinematic constraints, such as the turning radius of the mobile robot [6], the position of the robotic manipulator, and the speed and acceleration [7].

Different planning algorithms have their own characteristics. For mobile robots, some algorithms are based on grid maps, such as Dijkstra, A*, and D*. Each grid represents the location of free space or obstacles. These algorithms can find an optimal path with the smallest cost associated with the edges of the workspace structure. However, the workspace discretization resolution affects the rationality of the generated path. As the workspace size and the discretization resolution increase, computing time cost and memory requirements will increase exponentially, resulting in lower efficiency. The artificial potential field is a well-known path planning algorithm [8], in which the workspace is described using a potential field. A smooth path can be obtained by following the gradient of the established potential field. The local minimum and the non-reachable with the goal nearby obstacles are two important problems affecting these algorithms’ stability. Evolutionary algorithms such as genetic algorithms [9], differential algorithms [10], and ant colony algorithms [11] are also widely used in path planning. By mimicking biological evolutionary behavior, judging the optimal solution through adaptability function, and inheriting excellent features through selection algorithm, the generation path can be optimized. Recently, reinforcement learning has become an important issue in path planning research. It is a machine learning paradigm based on environment interaction, and can achieve the most strategic goals through trial and error and reward and punishment mechanisms. Q-learning is a typical path planning algorithm that uses reinforcement learning, which has self-learning ability and does not require a priori knowledge of the environment [12]. On this basis, many improved algorithms incorporating deep learning and neural networks have been proposed [13,14,15]. The advantage of these algorithms is that they can deal with high dimensional state space and action space, and thus be able to solve complex decision problems. However, learning in complex working environments is very time-consuming and the generated path is frequently not optimal. Local path planning algorithms, such as the dynamic window method (DWA), are sub-optimal methods based on predictive control theory. DWA can safely and effectively avoid obstacles in unknown environments, and has the characteristics of small computation, rapid response, and strong operability [16]. In the field of robot autonomous operations, environment mapping and localization is also an important topic, as it is a prerequisite for the robot to be able to travel on the planned paths. Simultaneous localization and mapping (SLAM) is mainly used to solve the problem of localization and map construction when robots move in unknown environments, and can assist robots in performing tasks such as path planning, autonomous exploration, and navigation [17]. Adaptive Monte Carlo localization (AMCL) is a probabilistic positioning system for robots in two-dimensional motion. Particle filters are used to track robot positions in known maps, which works well for a wide range of local positioning problems [18].

Sampling-based algorithms, such as the rapidly exploring random tree (RRT) [19] and the probabilistic roadmap [20], are widely used today. They have been demonstrated to offer highly effective performance, especially in high-dimensional and large-scale workspaces. The RRT algorithm constructs a random search tree from the initial state and incrementally extends the tree to the target state by continuously sampling waypoints. The generated path consists of a series of consecutive state nodes. Due to the lack of an optimization process, the generated path is not optimal and contains a large number of redundant nodes. Subsequently, a variety of RRT variants such as RRT* [21], RRT*-Connect [22], and Informed RRT* [23] have been proposed and extensively studied to improve path planning performance. RRT* is a significant improvement on the RRT algorithm. It does not directly connect the new sampling node to the nearest node on the tree, but introduces a cost value to optimize the selection of the parent node to achieve a near-optimal solution. It has been proved that the generated path converges to the optimal solution as the sampling nodes tend to infinity [21]. RRT*-Connect grows two trees incrementally simultaneously, one from the start state and another from the goal state. The two trees try to find a connection to obtain a path solution. When the target is in a narrow space or hidden behind an obstacle, it can effectively improve the path search efficiency. To accelerate the convergence, Informed RRT* limits the search area to a subset of the workspace to return a near-optimal solution quickly.

Recently, more and more optimized versions of RRT have been proposed. Qureshi and Ayaz [24] incorporated the artificial potential field algorithm in RRT*. It can greatly reduce the number of iterations, thus improving the memory utilization rate and accelerating the convergence speed. In order to solve the optimal path planning problem in narrow channels, Huang et al. [25] proposed adaptive informed RRT* (AI-RRT*), which can shorten the path length and asymptotically converge to the optimal solution. Wang et al. [26] proposed an elastic band-based rapidly exploring random tree (EB-RRT), which can achieve real-time optimal motion planning for the mobile robot in the dynamic environment. For path planning with kinematic constraints, the parent node selection and rewiring need to satisfy the constraint [27]. Moon and Chung [28] proposed a dual-tree rapidly exploring random tree (DT-RRT). It is composed of a workspace tree and a state tree. The workspace tree search takes is set in the target workspace without considering robot kinematics. Then the path is generated by the extension of the state tree under kinematic constraints. Wang [29] presented a kinematic constrained bi-directional rapidly exploring random tree (KB-RRT*), in which an efficient branch-pruning strategy is proposed to find a better parent node. Kuwata et al. [30] used closed-loop prediction in the RRT algorithm to enable the robot to track paths with kinematic constraints. Hu et al. [31] proposed an extended RRT algorithm to plan the motion of wheeled robots under kinematic constraints. The algorithm uses a path deformation strategy to shift the waypoint away from obstacles to generate short and smooth trajectories.

One problem that should also be noted is that the state of a robot in a workspace includes position and orientation information. In RRT-based algorithms with kinematic constraints, when the position distance and orientation difference between a new node and the target are both smaller than the two set thresholds, the target is considered to be connected [29]. One important reason for this is that, under kinematic constraints, it is difficult for a new node to satisfy both the position and orientation of the target at the same time. The shortest path planning is a classical problem. It has received a lot of attention due to the path length being one of the most useful metrics in the application of mobile robots. The objective is to find the shortest collision-free path between two state nodes in a workspace. Bounded curvature is the fundamental constraint in the motion planning of a car-like robot. Dubins has proved the geometric form of the shortest path with minimum curvature in a 2-D space. Based on this theorem, scholars have further discovered the characteristics of the shortest curvature-constrained path. This greatly extends the length optimal problem under the curvature constraints and enriches the corresponding solution.

In general, RRT*-based algorithms are essentially local optimization methods. They explore the whole configuration space randomly and uniformly to guarantee probabilistic completeness, resulting in slow convergence. The sampling node has a significant influence on the rationality of the path. Currently, most research focuses on how to sample waypoints and how to connect a pair of waypoints, which are two key factors affecting the quality of the path. Under kinematic constraints, the optimization is mainly in the new node generation stage. When a full connection path is generated, the waypoint state (position and orientation) cannot be changed. It is time-consuming to perform parent node selection and rewiring for each sampling node, and it is also not practical to obtain an optimal path through infinite samples. Therefore, the motivation of this paper is to combine the path optimization method with RRT path planning, and then propose an effective post waypoint shift strategy that can converge the initial generated random path to the shortest.

The main contributions of this study can be summarized as follows:

(1) A random complete path generation method under kinematic constraints is proposed, which incorporates fast RRT growth and goal connection algorithms.
(2) An effective waypoint shift algorithm based on Dubins curves is proposed to optimize random paths to the shortest length.
(3) In dynamic environments, an avoidance method for unknown obstacles on the planning path is proposed.

The remainder of this paper is organized as follows. The details of the path planning procedure of the proposed approach are introduced in Section 2. The simulation results and analysis of the generated paths in different working workspaces are presented in Section 3. In Section 4, the convergence path is proved to be the shortest. Then, the path planning performance of the proposed method is compared with other RRT-based methods, and real-world experiments are presented to show the effectiveness of the method. Finally, the feature property of the proposed model is summarized in Section 5.

## 2. Proposed Method

This section introduces the proposed method in detail, including the RRT growth strategy, goal connection method, and waypoint shift algorithm. Then, an efficient unknown obstacle avoidance method is presented.

### 2.1. RRT Growth Strategy

Consider a kinematic model of a car-like mobile robot. The two rear wheels are fixed parallel to the robot body and allowed to roll or spin. The two front wheels can turn to the left or right to change the forward direction. The state of a robot in a 2-D Cartesian coordinates is defined as **S** = (*x*, *y*, *θ*), and the kinematic model is described by the following equation

(1) $$\begin{array}{c} \dot{x}=v\cdot \cos\varphi \cdot \cos\theta \\ \dot{y}=v\cdot \cos\varphi \cdot \sin\theta \\ \dot{\theta }=v\cdot \sin\varphi /l \end{array}$$

where *v* is the driving velocity of the rear wheels, *θ* is the orientation angle of the robot body with respect to the X-axis, *φ* is the steering angle of the front wheels, and *l* is the wheelbase.

Constrained by mechanical structure, the steering angle *φ* is bounded by |*φ*| ≤ *φ*_max_. Therefore, the turning radius *ρ* cannot be less than the minimum turning radius.

(2) $$\rho \geq \rho_{\min}=l/\tan\varphi_{\max}$$

The RRT path planning is a sampling-based approach. The basic principle is that a random state S_rand_ is selected from the state space. Then, an extended function selects the nearest node S_nearest_ already in the tree to connect the sampling state S_rand_. If there is no obstacle, a new node S_new_ is generated from S_nearest_ to S_rand_ with an incremental distance *ε*. This procedure is repeated until the shortest distance between the goal state S_goal_ and S_new_ is smaller than a threshold. An extended tree will be generated from the initial state of the robot S_init_ to the goal state S_goal_, and a collision-free path can be obtained. However, since the sampling point is random, the nearest node may not meet the kinematic constraints. It reduces the success rate of the sampling nodes and occupies a lot of computation resources and reduces computing efficiency. This problem is particularly prominent when the bounded front-wheel steering angle |*φ*| is small.

This paper proposes an improved RRT growth strategy. The main idea is to select the closest node that satisfies the kinematic constraints as the parent node and extends the tree. When a random sampling node is generated, the first step is to find the closest node to the sampling node in the existing tree. Then, starting from this node, a new node is generated in the direction of the sampling node with the step size of *ε*. If the new node satisfies the kinematic constraints and there are no obstacles on the path, this new node will be added to the tree. Otherwise, the closest node is infeasible, and the next closest node is searched from the remaining nodes to determine its feasibility. If all the nodes in the tree do not meet the requirements, the sampling node will be rejected and another sampling point will be regenerated.

**Figure 1:**
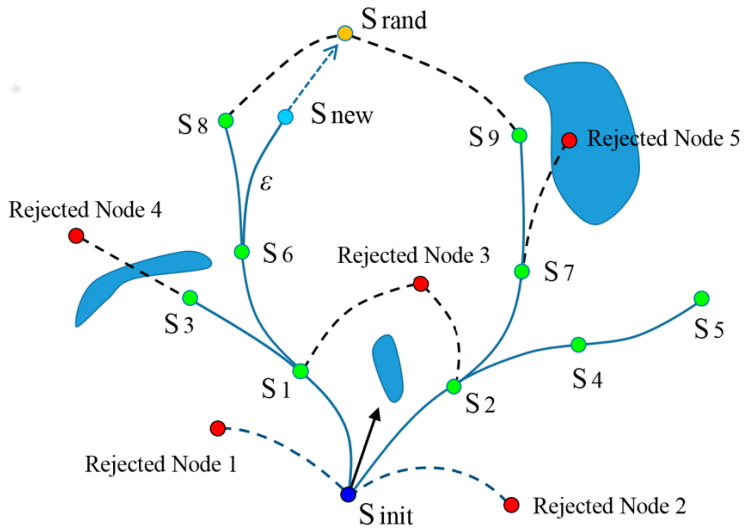
Demonstration of RRT growth method.

As shown in Figure 1, the initial state of the robot is S_init_. A sampling point node 1 is randomly generated. There is only the initial node S_init_ in the exploring tree and node 1 does not satisfy the kinematic constraints. Therefore, node 1 is rejected. Similarly, node 2 is also rejected. When the sampling point is S_1_, the kinematic constraints of the robot are satisfied from S_init_ to S_1_, so, S_1_ is added to the exploring tree and a branch is constructed. Suppose there are Nodes S_init_, S_1_ and S_2_ in the tree and the sampling point is node 3: Node 3 will be rejected, because neither S_1_ nor S_2_ satisfy the kinematic constraints, and there is an obstacle from S_init_ to node 3. Similarly, nodes 4 and 5 are both rejected. When the sampling point is S_rand_, the existing node closest to S_rand_ is S_8_, followed by S_9_. However, none of them satisfy the kinematic constraints. The third closest node S_6_ satisfies the kinematic constraints, and there is no obstacle within the distance *ε* from S_6_ to the S_rand_. Therefore, a new node S_new_ is generated and a new branch from S_6_ to S_new_ will be constructed. Through continuous sampling towards the goal with a certain probability, the node tree will gradually grow towards the goal area. Once the distance between the goal and the new node is less than the threshold *ε*_0_, the growth of the node tree will stop immediately.

The proposed RRT growth strategy has two characteristics. One is that it can make full use of all nodes in the tree to improve the success rate of the sampling points. When the bounded steering angle of the robot *φ*_max_ is small, it is difficult to find a parent node that satisfies the kinematic constraints in a certain area. A large number of sampling points will be rejected. Using the proposed method, any node in the RRT tree can satisfy the kinematic constraints, the sampling point will not be rejected, and a new branch will be generated. The increase in the success rate of the sampling points can effectively improve the growth rate of RRT to the target region, thus improving the efficiency of the initial path generation. The second is that the criterion for stopping tree growth is loose. With the growth of RRT, the orientation of the node near the target is random. It is impossible for the robot to reach the specified position and orientation from a random state in a small area, resulting in the failure to establish a complete path or the need to repeatedly move forward and backward to adjust the state. The proposed method does not consider this problem in the RRT growth process; instead, the goal connection method is subsequently proposed. This allows the tree to quickly find the target area and avoids being trapped near the goal due to kinematic constraints. The algorithm of the tree growth is shown in Algorithm 1.
**Algorithm 1.** RRT growth algorithm.**Function** RRT Growth **Inout:** S_init_, S_goal_, *ρ*_min_, *ε*, *ε*_0_, **Map** **Output:** Node tree **T**    1:   **T**_0_ ← {S_init_}    2:   **while** *k* ≤ LimitedNumber **do**    3:          S_rand_ ← RandomSample(Map)    4:          Distance *L*_i_ between S_rand_ and S_i_, ‖S_rand_ − S_i_‖, *_i_*_= 1 … *N*_    5:          **for** *i* = 1 ··· *N* **do**    6:                   Fine the smallest *L*_j_    7:                   S_new j_ = S_j_ + *ε*·(S_rand_ − S_j_)/‖S_rand_ − S_j_‖    8:                   Subpath S_j_ → S_new j_    9:                   **if** Feasibility (S_j_ → S_new j_) == *True* **then**    10:                        Extend(T, S_new j_)    11:                        **if** ‖S_new j_ − S_goal_‖ < *ε*_0_ **then**    12:                               **Reture T**    13:                               **end while**    14:                        **end if**    15:                **else**    16:                        Feasibility S_j_ == *False*    17:                **end if**    18:          **end**    19:   **end while**    20:   **return failure**

### 2.2. Goal Connection

**Figure 2:**
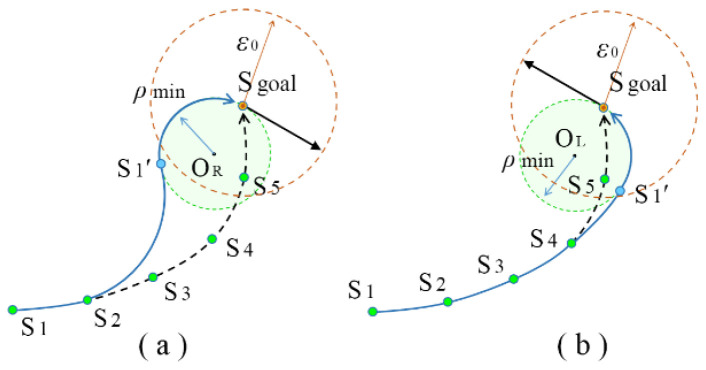
Demonstration of goal connection method. (**a**) clockwise (**b**) counterclockwise.

The tree growth described above can obtain a node path S_1_ → S_2_ → S_3_ → S_4_ → S_5_, whose distance between the last node S_5_ and goal S_goal_ is less than the threshold *ε*_0_, as shown in Figure 2. According to the RRT growth strategy, the kinematic constraints can be satisfied from S_1_ to S_5_. Both S_goal_ and S_5_ have their definite position and orientation information. This is needed to plan for the robot to reach the target position in the desired orientation.

This paper proposes a goal connection method based on the minimum turning radius. The first step is to find the minimum turning radius circle according to the goal state. Then, two arcs tangent to the circle can be constructed from a node in the path, and the two arcs rotate in opposite directions. A suitable arc needs to be chosen according to the orientation of S_goal_; Figure 2a is clockwise and Figure 2b is counterclockwise. The third step is to calculate the radius of the corresponding tangent arc from S_5_ to S_1_ sequentially. As shown in Figure 2a, when the radii of tangent arcs corresponding to Nodes S_5_, S_4_ and S_3_ are less than the minimum turning radius *ρ*_min_, they are rejected. When the radius of tangent arc S_2_ S_1_′ is greater than *ρ*_min_, the arc S_2_ S_1_′ is taken as the new path segment. The complete planning path is S_1_ → S_2_ → S_1_′ → S_goal_. The planned path fully satisfies the robot kinematic constraints and can reach the goal position at the desired orientation. Similarly, the complete path to the goal state in Figure 2b is S_1_ → S_2_ → S_3_ → S_4_ → S_1_′ → S_goal_. The algorithm of the goal connection is shown in Algorithm 2.
**Algorithm 2.** Goal connection algorithm.**Function** Goal Connection **Input: T**, S_goal_, *ρ*_min_ **Output:** complete path **P**    1:   The minimum turning radius circle, C _min_    2:   **for** *j* = M ··· 1 **do**    3:           Determine the arc tangent to the circle, C _j_    4:           Calculate the radius of C _j_, *ρ*_j_    5:           **if** *ρ*_j_ ≥ *ρ*_min_ **then**    6:                   Calculate the tangent point, S_1_^′^    7:                   **P** ←{ **T** _i = 1 : j_, S_1_^′^, S_goal_}    8:           **end if**    9:   **end**

### 2.3. Waypoint Shift Algorithm

The RRT growth under kinematic constraints is random and there is no rewiring optimization. Therefore, the initial generated path is not optimal. Here, an effective path optimization method that satisfies the robot kinematics constraints is proposed.

**Figure 3:**
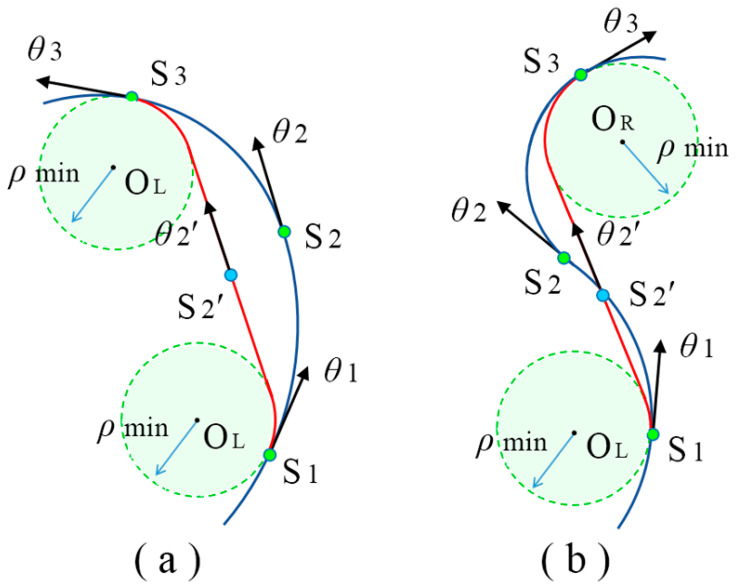
Demonstration of waypoint shift method. (**a**) the same turning direction (**b**) the opposite turning direction.

On two consecutive sub-paths, S_1_S_2_ and S_2_S_3_, the turning direction of the robot may be the same or the opposite, as shown in Figure 3. The first step is to find the two circles C _min S1_ and C _min S3_ with the minimum turning radius *ρ*_min_ corresponding to nodes S_1_ and S_3_. The second step is to determine the common tangents of the two circles. Obviously, there are four common tangents of the two circles. For the same turning direction, the outer common tangent that is close to nodes S_1_ and S_3_ is selected (Figure 3a). For the opposite turning direction, the inner common tangent is selected (Figure 3b). The third step is to find the center point S_2_′ of the common tangent. If there are no obstacles on the tangent path, the point S_2_′ will be used as a new node and the orientation is the direction of the common tangent θ_2_′. The optimized path becomes S_1_ → S_2_′ → S_3_. This new path is the shortest path that can satisfy the states of nodes S_1_ and S_3_ under the kinematic constraints. If there are obstacles on the tangent path, the state of node S_2_ remains unchanged, and the path is still S_1_ → S_2_ → S_3_. The fourth step is to select the next group of two consecutive sub-paths formed by S_2′_, S_3_ and the next node S_4_, and use the same method to optimize the path to obtain a new node S_3_′. From the initial state S_init_ to the goal state S_goal_, a new optimized path can be generated by adjusting the state of the intermediate nodes in turn. The essence is to use the Dubins curves satisfying the minimum curvature to optimize the path step by step. The algorithm of the waypoint shift is shown in Algorithm 3. Path optimization is a repeated iterative calculation process. As long as the state of the intermediate nodes does not change, an optimal path can be obtained.
**Algorithm 3.** Waypoint shift algorithm.**Function** Waypoint Shift, **Input:** complete path **P**, *ρ*_min_, map **Output:** optimal path **P**_optimal_    1:   **for** *p* = 1 ··· PathStep **do**
    2:           The minimum turning radius circles, C _min p_, C _min p+2_    3:           Direction of rotation of subpaths S_p_S_p+1_ and S_p+1_S_p+2_    4:           Determine the arc tangent to the circle, C _p,p+2_    5:           Center point of C _p,p+2_, S_p+1_^′^    6:           **if** Collide(S_p_→S_p+1_^′^→S_p+2_) == *False* **then**    7:               **P**_optimal p,p+2_ ← { S_p_, S _p+1_^′^, S _p+2_}    8:               else    9:               **P**_optimal p,p+2_ ← { S_p_, S _p+1_, S _p+2_}    10:       **end**    11: **end**

### 2.4. Unknown Obstacle Avoidance

**Figure 4:**
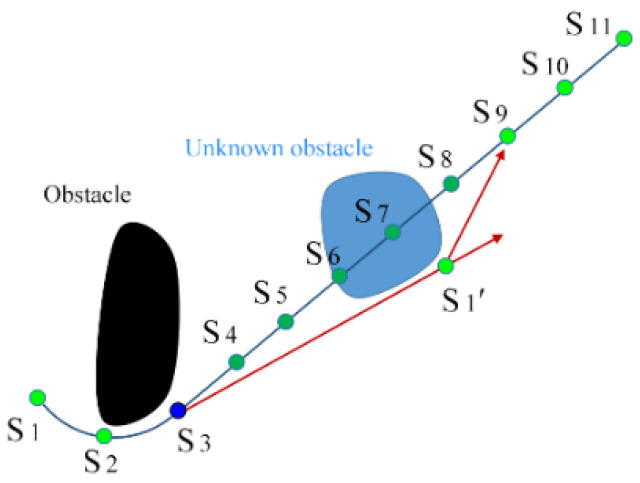
Demonstration of unknown obstacle avoidance.

The work environment is often dynamic. While the robot is moving, the on-board radar will monitor the surrounding environment in real time. As shown in Figure 4, this is assuming that the original optimized path is S_1_ → S_2_ → … → S_10_ → S_11_ are marked as the green point ‘•’. When the robot moves to node S_3_ is marked as the blue point ‘•’, it finds an unknown obstacle blocking the planned path. In this case, the robot needs to re-plan the path to bypass the obstacle.

When the edge of an unknown obstacle is detected, the robot will turn to the corner of the obstacle and travel in a straight line in a tangential orientation. When the robot bypasses the obstacle and reaches node S_1_′, it can find some original path nodes, S_8_ to S_11_, that are not occupied by the obstacle. It will use the goal connection method shown in Figure 2 to search the nearest node S_9_ that satisfies the kinematic constraints. The re-planned path becomes S_1_′→S_9_→S_10_→S_11_, since the path after node S_9_ is feasible when there are no new unknown obstacles. Using the proposed waypoint shift algorithm, the shortest path can be optimized in the current situation.

## 3. Simulations and Analysis

In this section, to verify the performance of the proposed method, the path planning simulations are carried out in an open workspace, a maze workspace, and a workspace cluttered with small obstacles. The initial random path generation and the path optimization are analyzed. Subsequently, one simulation is performed in a dynamic environment with unknown obstacles.

### 3.1. Open Workspace

**Figure 5:**
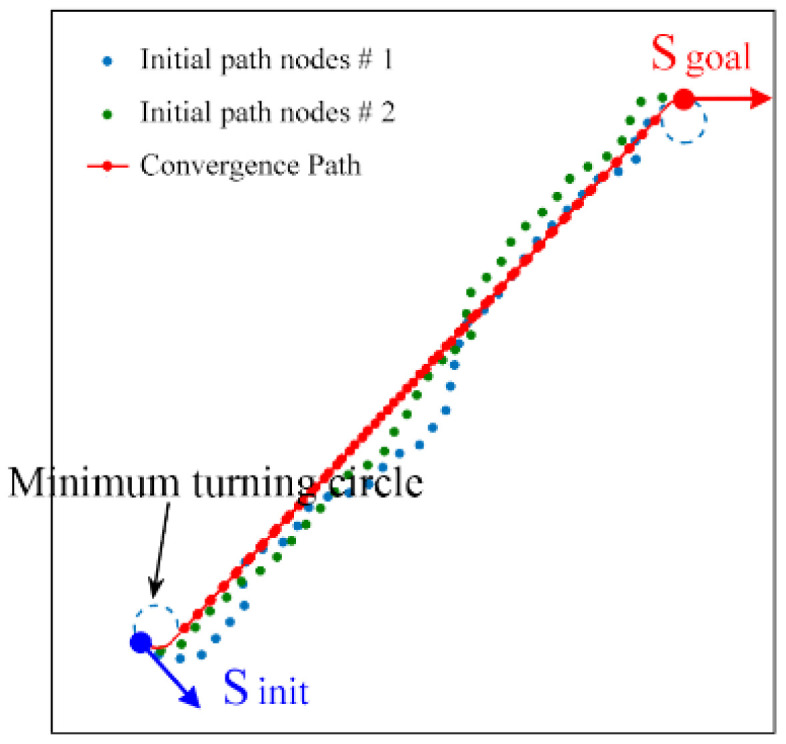
Path planning in an open workspace.

To demonstrate the basic characteristics of the proposed approach, the path planning is first carried out in an open workspace, as shown in Figure 5. It is assumed here that the minimum turning radius of the robot *ρ*_min_ is 12, the incremental distance *ε* is 12, and the threshold *ε*_0_ is 15. The size of the workspace is 400 × 400 (unit: pixel). The initial state S_init_ = (350, 50, π/4) and the goal state S_goal_ = (50, 350, π/2). The path planning is conducted twice, and two initial random paths are generated. Then, the proposed waypoint shift method is used to optimize the two random paths. The robot will turn counterclockwise along the minimum turning circle of the initial state **𝒞**_init_. Then, it moves straight along the inner tangent of **𝒞**_init_ and the minimum turning circle of the goal state **𝒞**_goal_, and finally, it turns clockwise to the goal state along **𝒞**_goal_. Although the initial paths are different, the optimized paths can gradually converge to the same line, as marked with the red point ‘•’.

### 3.2. Maze Workspace

**Figure 6:**
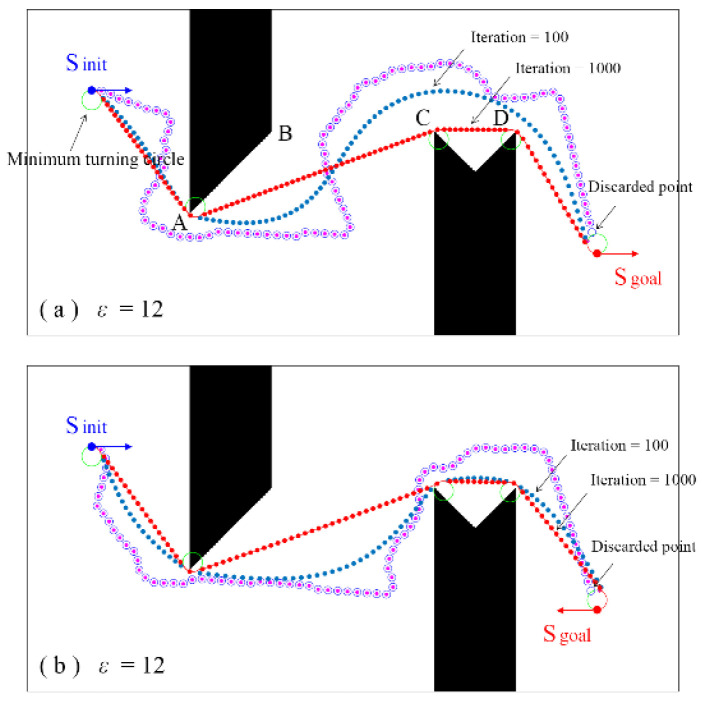

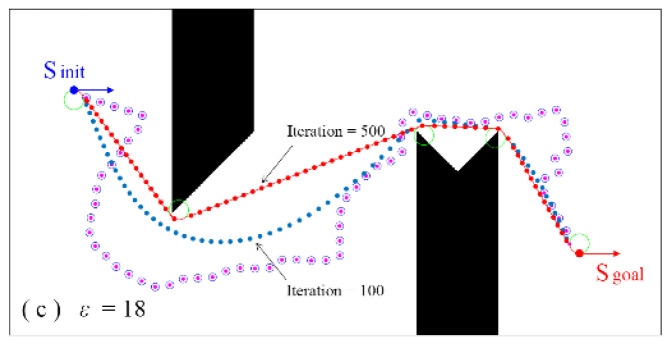
Path planning in a maze workspace. (**a**) *ε* = 12 (**b**) *ε* = 12 (**c**) *ε* = 18.

Figure 6 shows a maze workspace with a size of 800 × 400. There is a spike obstacle and a concave obstacle. The initial state of the robot is S_init_ = (100, 80, π/2).

When the goal state is S_goal_ = (300, 700, π/2), Figure 6a shows the path planning result. In the RRT growth stage, the paths to the goal area are obtained, which are marked as blue empty circles ‘○’. Then, the tangent point S_1_′ to the minimum turning radius arc is calculated according to the goal connection method, and the complete path is generated, which is marked as the pink point ‘•’. Due to the constraint of the minimum turning radius, some nodes close to the goal are discarded. Then, the proposed waypoint shift algorithm is used to optimize the initial random paths. Path optimization is an iterative process from the initial state to the convergence state. With the increase in the iterative calculation, the path will become smoother. When the number of iteration calculations is 100, the obtained path is marked as the blue point ‘•’. By further increasing the number of iteration calculations, the path will gradually converge, which is marked as the red point ‘•’. The robot adjusts its forward direction along the arc with the minimum turning radius from the initial state S_init_, travels in a straight line to the tangent point of the arc with the minimum turning radius at point A, and then uses the same method to bypass points C and D to reach the goal state S_goal_. It is a concave obstacle between points C and D. The proposed optimization method does not fall into this trap, but selects the shortest path to bypass it. This demonstrates that the proposed waypoint shift method has good convergence, and the optimized path does not depend on the initial random path.

When the goal state is S_goal_ = (300, 700, 3π/2), the generated initial complete path and the optimized path are shown in Figure 6b. It can be found that the convergence path uses the same method to bypass obstacles, and the difference is that it turns counterclockwise along the minimum turning radius arc of S_goal_ to reach the goal position with the desired orientation of 3π/2, rather than clockwise as shown in Figure 6a.

### 3.3. Small Obstacle Avoidance

**Figure 7:**
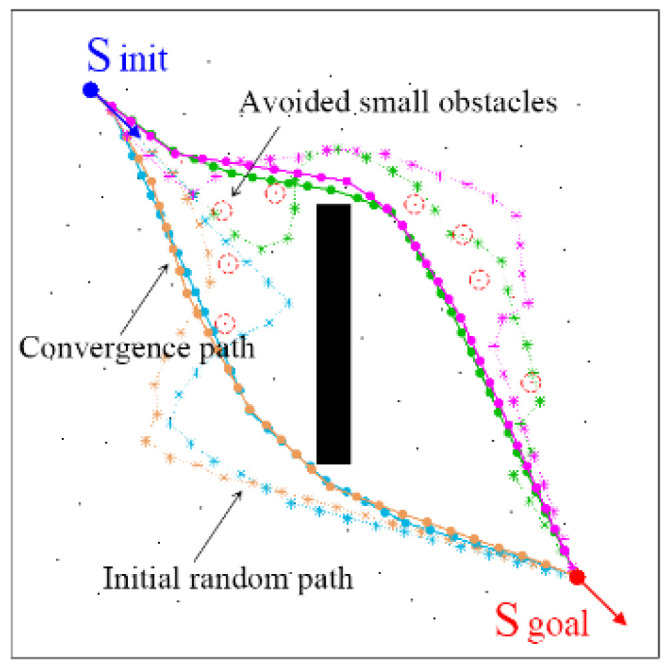
Small obstacle avoidance on path optimization.

Obstacle avoidance performance is important in evaluating models. Small obstacles often appear in the actual workspaces. To verify the influence of small obstacles on path optimization, simulations are carried out in a complex workspace. As shown in Figure 7, the workspace has a size of 400 × 400 with a rectangular obstacle in the middle area. Some small obstacles are randomly scattered. The initial state and the goal state are S_init_ = (100, 80, π/4) and S_goal_ = (300, 700, π/4), respectively. The initially planned path may choose to bypass the rectangular obstacle from above or below to reach the goal state. Using the proposed waypoint shift method, the path can avoid small obstacles and reach the optimal paths. The avoided small obstacles are marked as red dots ‘▪’. This is because the waypoint shift only analyzes whether there are obstacles in the optimized path. As shown in Figure 3, obstacles in the area surrounded by the paths S_1_S_2_S_3_ and S_1_S_2_′S_3_ have no effect on node shift. However, when the obstacle is exactly on the optimal path, the waypoint shift is not feasible. Because the position and number of nodes in the initial paths are different, the optimized paths will be slightly different.

### 3.4. Dynamic Environment

**Figure 8:**
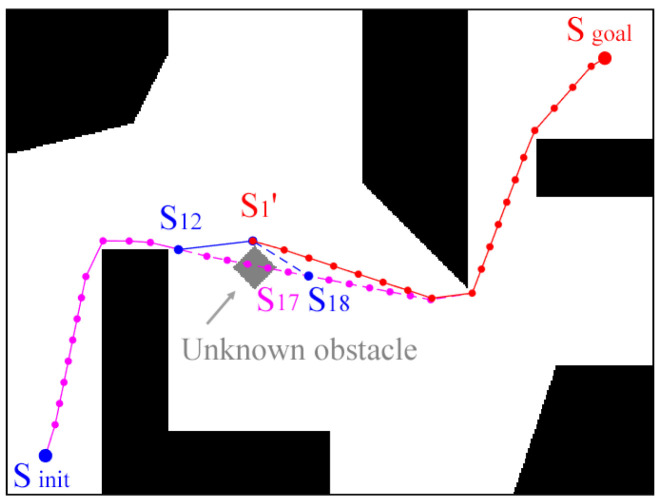
Path planning in a workspace with an unknown obstacle.

As shown in Figure 8, it is assumed here that there is a convergence path under a known environment, marked as the pink point ‘•’. When the robot moves to node S_12_, it detects an unknown obstacle on the planned path. Then, the robot changes its original path and drives to point S_1_′ on the edge of the obstacle. After passing point S_1_′, the robot finds the original planned path after node S_17_. Since node S_17_ does not meet the kinematic constraint, point S_18_ is chosen as the path node connecting point S_1_′. A new convergence path is generated through the waypoint shift, which is marked as the red point ‘•’. The connection method of points S_1_′ and S_18_ is the same as the goal connection method given in Figure 2. Since the path from S_1_′ to S_18_ satisfies the minimum curvature, the convergence path is also composed of segmented Dubins curves. When no new unknown obstacles are found, the original path nodes after node S_18_ are feasible. This makes it possible to utilize the prior knowledge without performing a new path search. Therefore, this re-planning method is efficient. It should also be noted that there is uncertainty in avoiding an unknown obstacle because the shape of the obstacle is variable. In some worst-case scenarios, when the robot avoids unknown obstacles, it cannot find the original path nodes, or none of the original path nodes satisfy the kinematic constraints. This means that the original path behind the obstacle becomes infeasible, which requires a re-RRT path planning operation to generate a new path. In general, the occurrence of this case is random and accidental, and it is mainly related to the shape of the obstacle and the location in the working environment.

## 4. Discussion and Experiments

In this section, it is proved that the convergence path is the shortest. Then, the influence of model parameters on the path convergence performance is analyzed. To illustrate the characteristics of the proposed method, the path planning results of the proposed method and previous RRT-based algorithms are compared. Finally, one experiment is carried out in real-world environments.

### 4.1. Shortest Path Proof

In an open workspace, it can be proved that the convergence path optimized using the waypoint shift is the optimal shortest path. As is well-known, the Dubins curve is the shortest path connecting two states in the plane under curvature constraint. Therefore, the idea is to prove that the convergence path coincides with the Dubins curves.

**Proof.**  Assume that the obtained convergence path **P** is S_init_ → S_1_ → ··· → S_p_ → S_goal_. We can prove by contradiction that nodes S_1_ to S_p_ form a straight line with the same orientation. According to the waypoint shift illustrated in Figure 3, node S_1_ is always on the tangent of C _min Sinit_ with the orientation of *θ*_1_. Then, nodes S_1_, S_2_, and S_3_ are consecutive nodes. If they are not in a straight line or the orientation angles are different. There must be a common tangent between C _min S1_ and C _min S3_, and nodes S_2_ and S_2_′ cannot coincide. Thus, the path does not converge and the waypoint shift will not stop. This contradicts the assumption that the path is convergent. If there are three consecutive nodes S_i–1_, S_i_, S_i+1_ ⸦ {S_1_, S_2_, ··· S_p_}, they also form a straight line with the orientation of *θ*. This is due to the fact that node S_p_ is always on the tangent of C _min Sgoal_ with the orientation of *θ*_p_. Therefore, the orientation of the straight line, *θ* = *θ*_1_ = ··· = *θ*_p_, can only be the common tangent direction of C _min Sinit_ and C _min Sgoal_. The convergence path P coincides with the Dubins curves. In the environment with obstacles, the waypoint shift will transfer node S_i_ to the position closest to the obstacle. The straight path in the open workspace will be tangent to the minimum radius circle C _min Si_ and bypass the obstacle. Therefore, the full path is composed of segmented Dubins curves, as shown in Figure 5. □

### 4.2. Parameter Sensitivity

Parameter sensitivity is an important index for model evaluation. In the proposed method, the incremental distance *ε* is a major parameter that affects the path growth rate and the number of nodes in the path. When *ε* is set to 18, Figure 6c shows the path planning and optimization results. It can be seen that the incremental distance *ε* has little effect on the convergence path, and the obtained convergence paths are essentially consistent. However, *ε* will affect the path convergence rate. The reason for this is mainly related to the waypoint shift method. As shown in Figure 3, the adjustment distance from S_2_ to S_2_′ depends not only on the orientation angles of the three consecutive nodes S_1_, S_2_, and S_3_, but also on the distance between them. As *ε* increases, the displacement of each node adjustment will also increase, which can accelerate the convergence rate of the initial random path. It should also be pointed out that this convergence rate is related to the initial path. The initial generated path is random. The more it deviates from the convergence path, the more iterative calculations are required.

### 4.3. Comparative Discussion

In summary, compared to the existing RRT-based approach, the main characteristic of the proposed method is that it post optimizes the initial random path to the shortest path under kinematic constraints.

Since the RRT method was proposed, path optimization has been a focus of this research field. RRT* is the most successful method, in which parent node selection and rewiring are performed. The common problem is that once a new node is added to the tree, its state cannot be changed again, and this is also the main reason why the RRT* algorithm can only obtain near-optimal solutions, especially under kinematic constraints. In this paper, a novel waypoint shift method is proposed. It can optimize the complete path after it is generated, rather than at the new node generation stage. The optimized path has good convergence and robustness, and can effectively avoid the interference of small obstacles. An important feature of the post waypoint shift is that the optimization is limited to the nodes of the path, rather than all exploring nodes on the tree. Taking Figure 6a as an example, to generate an initial path, the total exploring nodes on the tree usually need to number more than 450, and the number of nodes on the path is only about 90. The RRT* algorithm needs to perform parent node selection and rewiring optimization during each node generation [19,28,29]. This means that a lot of node optimization calculations are redundant. With the increase in the size and complexity of the environment, the number of sampling points will increase exponentially. This calculation workload is enormous and time consuming. The proposed method can avoid a lot of unnecessary calculations, thereby improving the efficiency of path optimization.

Another feature is the initial path generation method. In current RRT-based algorithms, when the permitted steering angle of the robot is small, a large proportion of random sampling points cannot meet the kinematic constraints in this way. The workspace is shown in Figure 6a, and the minimum turning radius of the robot *ρ*_min_ is 6. The average number of sampling points is about 2600, and the average number of nodes in the exploring tree is about 700. The success rate is about 27%. Using the proposed tree growth strategy, the activity of each node on the tree can be motivated to maximize the success rate of the sampling points. The average number of sampling points and the number of nodes in the exploring tree is about 850, and the corresponding success rates can be increased to about 57%. In addition, the number of nodes in the paths is reduced accordingly. This will significantly improve the efficiency of the initial random path generation, and the subsequent path optimization. This advantage is present even in an open workspace.

### 4.4. Real-World Experiment

**Figure 9:**
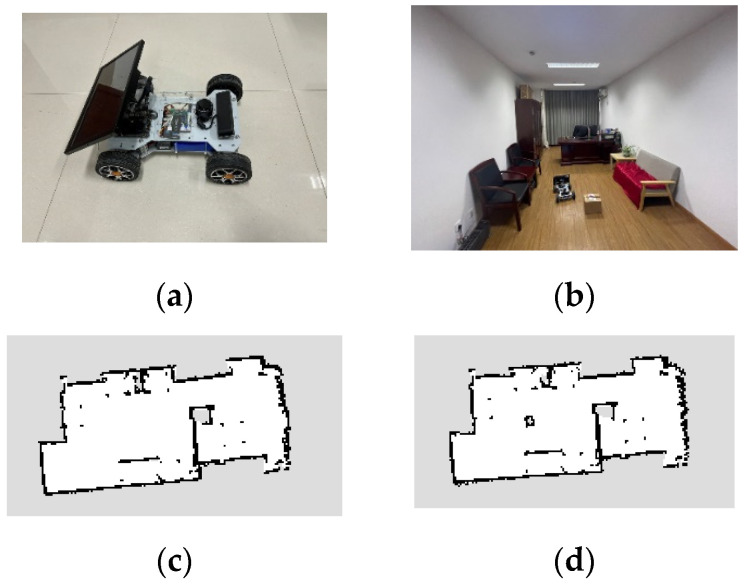
Real-world experiment situation. (**a**) the robot body (**b**) the test situation (**c**) the prior workspace information (**d**) updating the workspace information.

To verify the effectiveness of the proposed method, the experiment was carried out in an office environment using an AKM R550 mobile robot by WHEELTEC in Guangdong, China. The robot is equipped with a Jetson Nano processor, and a positioning and navigation system, as shown in Figure 9a. The size of the robot is 440 mm × 350 mm and the wheelbase is 300 mm. Limited by the steering angle of the front wheels, the minimum turning radius is about 0.5 m. Driven by two MG513 DC deceleration motors, the movement velocity of the robot is set to 0–0.2 m/s. A Radon N10 LiDARby WHEELTEC in Guangdong, China, is mounted on the front of the robot. Its measuring radius is 25 m, scanning frequency is 10 Hz and angular resolution is 0.8°. The actual office workspace size is 6.8 m × 3.1 m, and the test situation is shown in Figure 9b.

**Figure 10:**
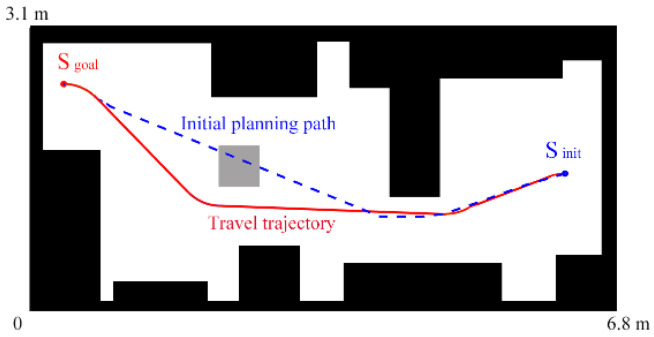
Illustration of the robot’s trajectory.

The robot first scans the office to obtain prior workspace information, as shown in Figure 9c. Then, an unknown obstacle occurs on the planned path. Its shape and location are unknown. When the on-board LiDAR detects an unknown obstacle while traveling, it scans the boundary of the obstacle in real time and updates the workspace information, as shown in Figure 9d. If the unknown obstacle is on the pre-planned path, the robot will re-plan its path and march to the target state according to the profile occupation. Figure 10 presents the initial planning path and the actual traveling path of the robot center. This shows that the proposed method is effective in practical applications.

## 5. Conclusions

In this paper, an RRT algorithm with an effective post waypoint shift was proposed. It selects the nearest existing node that satisfies the kinematic constraints as the parent node, thereby improving the success rate of the sampling nodes. Then, the goal connection method based on the minimum turning radius arc is used to generate an initial random path. Finally, waypoint shift iteration is performed under kinematic constraints to achieve the optimal path. The simulation results indicate that the proposed method can present a better solution compared with other RRT-based algorithms in terms of path length and convergence. Several points are worth noting about the proposed model:

(1) A parent node selection and RRT growth strategy is proposed, which can improve the success rate of sampling nodes. Under kinematic constraints, a goal connection method is proposed, which enables the robot to reach the goal position with the desired orientation.
(2) An effective post waypoint shift algorithm is proposed to obtain the optimal path. It does not perform parent node selection and rewiring for each sampling node during RRT growth, but performs optimization after establishing an initial random path.
(3) The proposed waypoint shift method can effectively avoid small obstacles to obtain the convergence path. The obtained path is composed of Dubins curves, which is in the sense of the shortest path in an open workspace and the local optimal path in a multi-channel workspace.
(4) An efficient method to avoid unknown obstacles is proposed, which makes it possible to utilize the information of the original path instead of re-planning the path. The generated path can still converge to the shortest one in the current state.
(5) The proposed method is effective and robust. The initial random path, and model parameters such as incremental distance have little influence on the convergence path; however, they will have some influence on the convergence speed. Compared with the existing RRT-based algorithms, it has significant advantages in terms of the sampling point number, the tree node number, and the path node number.

Many facets remain for future investigation. The proposed convergence path will choose the minimum turning radius to achieve a shorter path. The curvature of the path is not continuous, and the robot needs to bypass the obstacle at a low speed. We are working on building transition curves to make the path more comfortable. Furthermore, combining the proposed method with APF and other sampling methods to further improve the RRT growth rate and convergence speed would also be worth studying.

## Data Availability

The data are contained within the article.
